# Ultra-high dose rate (FLASH) carbon ion irradiation inhibited immune suppressive protein expression on Pan02 cell line

**DOI:** 10.1093/jrr/rrae091

**Published:** 2024-12-27

**Authors:** Shohei Katsuki, Kazumasa Minami, Karin Oniwa, Masashi Yagi, Shinichi Shimizu, Noriaki Hamatani, Masaaki Takashina, Tatsuaki Kanai, Kazuhiko Ogawa

**Affiliations:** Department of Medical Physics and Engineering, Osaka University Graduate School of Medicine, 1-7 Yamadaoka, Suita, Osaka, 565-0871, Japan; Department of Medical Physics and Engineering, Osaka University Graduate School of Medicine, 1-7 Yamadaoka, Suita, Osaka, 565-0871, Japan; Department of Medical Physics and Engineering, Osaka University Graduate School of Medicine, 1-7 Yamadaoka, Suita, Osaka, 565-0871, Japan; Department of Carbon Ion Radiotherapy, Osaka University Graduate School of Medicine, 2-2 Yamadaoka, Suita, Osaka, 565-0871, Japan; Department of Carbon Ion Radiotherapy, Osaka University Graduate School of Medicine, 2-2 Yamadaoka, Suita, Osaka, 565-0871, Japan; Department of Medical Physics, Osaka Heavy Ion Therapy Center, 3-1-10 Otemae, Chuo-ku, Osaka-city, Osaka, 540-0008, Japan; Department of Medical Physics, Osaka Heavy Ion Therapy Center, 3-1-10 Otemae, Chuo-ku, Osaka-city, Osaka, 540-0008, Japan; Department of Medical Physics, Osaka Heavy Ion Therapy Center, 3-1-10 Otemae, Chuo-ku, Osaka-city, Osaka, 540-0008, Japan; Department of Radiation Oncology, Osaka University Graduate School of Medicine, 2-2 Yamadaoka, Suita, Osaka, 565-0871, Japan

**Keywords:** FLASH, ultra-high dose rate, carbon ion beam, calreticulin (CRT), PD-L1

## Abstract

Recently, ultra-high dose rate (> 40 Gy/s, uHDR; FLASH) radiation therapy (RT) has attracted interest, because the FLASH effect that is, while a cell-killing effect on cancer cells remains, the damage to normal tissue could be spared has been reported. This study aimed to compare the immune-related protein expression on cancer cells after γ-ray, conventionally used dose rate (Conv) carbon ion (C-ion), and uHDR C-ion. B16F10 murine melanoma and Pan02 murine pancreas cancer were irradiated with γ-ray at Osaka University and with C-ion at Osaka HIMAK. The dose rates at 1.16 Gy/s for Conv and 380 Gy/s for uHDR irradiation. The expressed calreticulin (CRT), major histocompatibility complex class (MHC)-I, and programmed cell death 1 ligand (PD-L1) were evaluated by flow cytometry. Western blotting and PCR were utilized to evaluate endoplasmic reticulum (ER) stress, DNA damage, and its repair pathway. CRT, MHC-I on B16F10 was also increased by irradiation, while only C-ion increased MHC-I on Pan02. Notably, PD-L1 on B16F10 was increased after irradiation with both γ-ray and C-ion, while uHDR C-ion suppressed the expression of PD-L1 on Pan02. The present study indicated that uHDR C-ion has a different impact on the repair pathway of DNA damage and ER than the Conv C-ion. This is the first study to show the immune-related protein expressions on cancer cells after uHDR C-ion irradiation.

## INTRODUCTION

Radiation therapy (RT) kills cancer cells via DNA damage. This phenomenon is caused by not only irradiation that excites DNA molecules and damages it, but also reactive oxygen species (ROS) produced by RT [1]. Recently, in addition to X-ray, carbon ion (C-ion) has been utilized for cancer treatment because of its favorable physiological and biological features; dose-concentration with high accuracy, and high cell-killing effect, respectively [2, 3]. These days, several studies reported ultra-high dose rate irradiation (> 40 Gy/s, uHDR; FLASH) of X-ray, electron, and proton can spare the damage to normal tissue but retain its cell-killing effect on cancer cells, called FLASH effect [4–6].

However, the impact of uHDR carbon ion (C-ion) irradiation on cancer immunogenicity is still uninvestigated. One of the reasons is that the number of facilities that can irradiate uHDR C-ion is limited worldwide and the irradiation field was not large enough to conduct several basic biological experiments, therefore it was a challenge to obtain enough cells for the experiments. Recently, in Osaka HIMAK, the uHDR C-ion irradiation field achieved $16\times 16$ [mm^2^], enough to cover a single well in a 24-well plate [7, 8]. Due to this achievement, approximately $2\times{10}^5$ cells can be obtained and is enough to analyze the protein expression by flow cytometry. To investigate the impact of uHDR C-ion irradiation on immunogenicity, calreticulin (CRT), major histocompatibility complex class-I (MHC-I), and programmed cell death 1 ligand (PD-L1) were selected [9]. CRT, an endoplasmic reticulum-resident protein and ER stress induces its translocation to the cell membrane, works as an ‘eat me signal’ and enhances phagocytosis by dendritic cells (DCs), then DCs present tumor-specific antigens to T cells and exert antitumor immune response [10, 11]. MHC-I on cancer cells presents its unique antigen therefore, activated T cells can recognize and attack it [12–14]. PD-L1, a ligand of the PD-1 receptor, conversely leads to T cell anergy [15–17].

In the present study, we showed that uHDR C-ion mainly has a similar impact on the expression of immunogenic protein, however, PD-L1 expression on a murine pancreas cancer cell was suppressed by uHDR C-ion compared with Conv C-ion irradiation.

## MATERIAL AND METHOD

### Cell lines

B16F10 murine melanoma and Pan02 murine pancreatic adenocarcinoma cell lines were purchased from RIKEN Cell Bank (Japan) and the National Institutes of Health (MD, USA), respectively. Both cells were cultured with Dulbecco Modified Eagle Medium (DMEM) supplemented with 10% FBS, 5 mM penicillin/streptomycin, and L-glutamine in an incubator under 37°C with 5% CO_2_ atmosphere.

### Irradiation

One day before irradiation, 5 $\times$10^5^ cells were seeded in a 24-well plate with 1.4 mL of medium at Osaka University. Cells were irradiated with C-ion with linear energy transfer (LET) 50 keV/$\mathrm{\mu} \mathrm{m}$ under the conditions of 1.16 Gy/s (Conventional; Conv) and 380 Gy/s (uHDR) [7, 8]. After irradiation, the plates were transported to Osaka University, and the medium was replaced and incubated for 2 days until flow cytometric analysis. Irradiation with γ-ray was conducted with the Gammacell 40 Exactor (NORDION, CANADA) at the same time as the C-ion irradiation.

### Colony formation assay

Irradiated cells were collected and seeded into 60 mm dishes. Approximately two weeks after culturing, the cells were fixed with formalin and stained with crystal violet solution. The number of colonies was counted and the survival fraction (SF) in each dose was calculated. Then, SF curves were fitted with the Linear-Quadratic (LQ) model as follows; $SF=\exp \left(-\alpha D-\beta{D}^2\right)$, where D is the dose and both a and b are constant.

### Flow cytometry

Collected cells were stained for 1 hour with the following antibodies diluted with FACS buffer (PBS/10% FBS and 0.5 mM EDTA); CRT (BIS-BS-5913R-FITC, Bioss), PD-L1 (12–5952-81, eBioscience), and MHC-I (H-2K^b^: 17–5958-82, eBioscience). After staining, cells were washed with FACS buffer, transferred into a flow tube, then proceeded with flow cytometric analysis by FACS Canto II (BD). The data was analyzed with Flow Jo® ver. 10.8.9 (BD).

### RNA preparation and quantitative RT-PCR

RNA samples were collected from cultured cells according to the protocol of the RNeasy Mini kit (QIAGEN, Germany) and reverse-transcribed to cDNA. Quantitative polymerase chain reaction with reverse transcription (qRT-PCR) was performed with PowerSYBR Green PCR Master Mix (Thermo Fisher Scientific). The gene sequences are below:

*Brca2* Fw: ATGCCCGTTGAATACAAAAGGA*,* Rv: ACCGTGGGGCTTATACTCAGA.

*Topbp1* Fw: CAGGATTGTTGGTCCTCAAGTG, Rv: CAGGATTGTTGGTCCTCAAGTG.

*Gapdh* Fw: GTTGTCTCCTGCGACTTCA, Rv: GGTGGTCCAGGGTTTCTTA.

The ${2}^{-\Delta \Delta Ct}$ method was used to evaluate the fold expression change.

### Western blotting

Cells were lysed with Pierce™ RIPA buffer (Thermo Fisher Scientific) with protease/phosphatase inhibitor. The supernatant was obtained by centrifugation at 12,000 rpm for 15 min at 4°C. The protein concentrations were measured with Pierce™ BCA Protein Assay Kits (Thermo Fisher Scientific). Each sample was mixed with 4X Laemmli Sample Buffer (BIO-RAD, USA) containing 10% $\beta$-ME (Thermo Fisher Scientific), incubated at 95°C for 5 min, and then placed in a gel. The electrophoresis was conducted at 75 kV for 20 min, followed by 120 kV for 40 min, then proteins were transferred to a PVDF membrane. The membrane was stained with anti-phosphorylated eIF2a (#3597, CST, USA), anti-gH2AX (#9718, CST), and anti-GAPDH (#97166, CST).

### Statistic

Data was obtained by at least three independent experiments in every flow cytometry experiment. Tukey’s honestly significant difference test was used to compare the expressions. *P*-values were adjusted by the Bonferroni method for multiple comparisons. A significant difference was defined as *P* < 0.05.

## RESULTS AND DISCUSSION

**Fig. 1:**
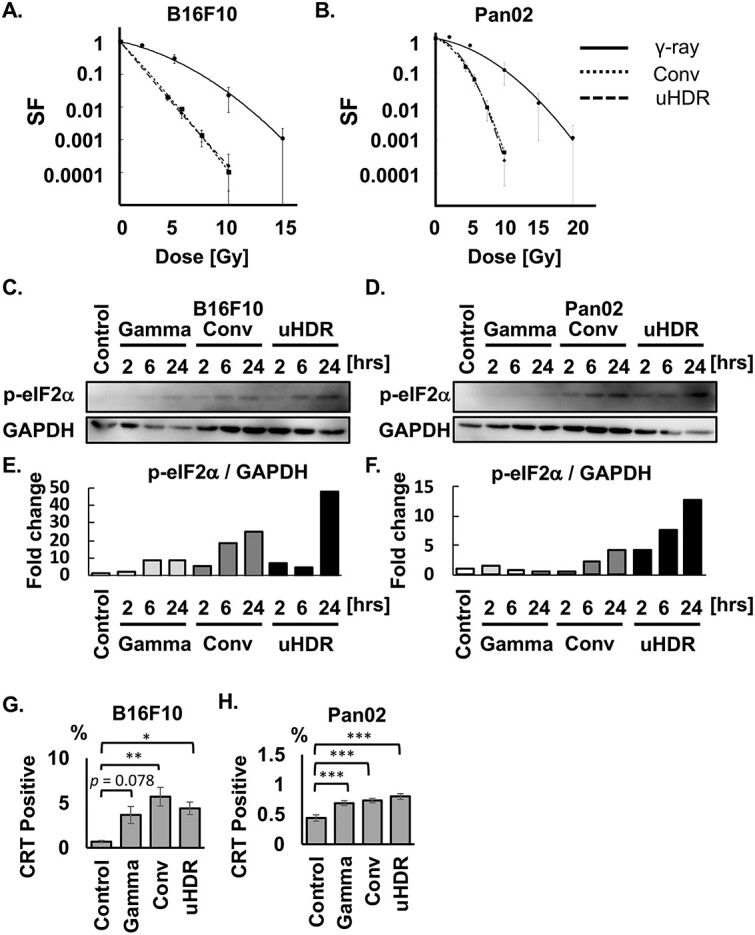
The impact of uHDR C-ion irradiation on the ER. (A, B) SF of B16F10 (A) and Pan02 (B) calculated by colony formation assay were shown. Solid line, dot line, and dashed line indicate γ-ray, Conv, and uHDR C-ion, respectively. For each SF curve, we conducted three independent experiments. Data are indicated mean ± S.E. The curves were fitted with an LQ model. (C-F) Phosphorylated eIF2a and GAPDH in control at the indicated time pot-irradiation in B16F10 and Pan02 were shown, and the ratios of these proteins’ expression were calculated. (G, H) Calreticulin-positive populations on B16F10 (G) and Pan02 (H) at 48 hrs after irradiation were shown. ^*^P < 0.05, ^**^P < 0.01, ^***^P < 0.001.

Colony formation assay was conducted to determine the dose of γ-ray and C-ion used in the following experiments. For both B16F10 and Pan02 cancer cell lines, the ‘FLASH effect’ was not observed at least 10 Gy or less, and the SF curves under both Conv and uHDR conditions in both cell lines were almost identical (Fig. 1A, B). The calculated relative biological effect at 1% survival (RBE_1_) was approximately 2.2 in both cell lines shown in Fig. 1. Because of the machine setting, we could choose several specific doses of uHDR irradiation, therefore, we selected 5.65 Gy of carbon ion under both Conv and uHDR and 11.1 Gy with γ-ray as the almost same biological effective dose (BED) in the following experiments.

To investigate the difference in impacts of γ-ray and C-ion under both Conv and uHDR conditions on the ER, phosphorylated eukaryotic translation initiation factor (eIF2a) was evaluated at various time points post-irradiation within 24 hrs. When ER stress occurs, eIF2a is phosphorylated into p-eIF2a. Western blotting revealed that p-eIF2a was expressed after C-ion irradiation regardless of dose rate on B16F10 and Pan02 cells (Fig. 1C and D). While the expression ratio (p-eIF2a / GAPDH) showed that uHDR had greater impacts on ER than Conv in both cells (Fig. 1E and F), CRT expressions on cancer cells were similarly increased 48 hrs post-irradiation (Fig. 1G and H). CRT has been reported to be translocated to the cell membrane after ER stress [9, 18]. Although the expression analysis of p-eIF2a revealed that uHDR C-ion induced the highest expression 24 hrs post-irradiation (Fig. 1E and F), uHDR induced the CRT as high as Conv and γ-ray at 48 hrs post-irradiation (Fig. 1G and H). These results indicate that uHDR can enhance DCs’ phagocytosis.

**Fig. 2:**
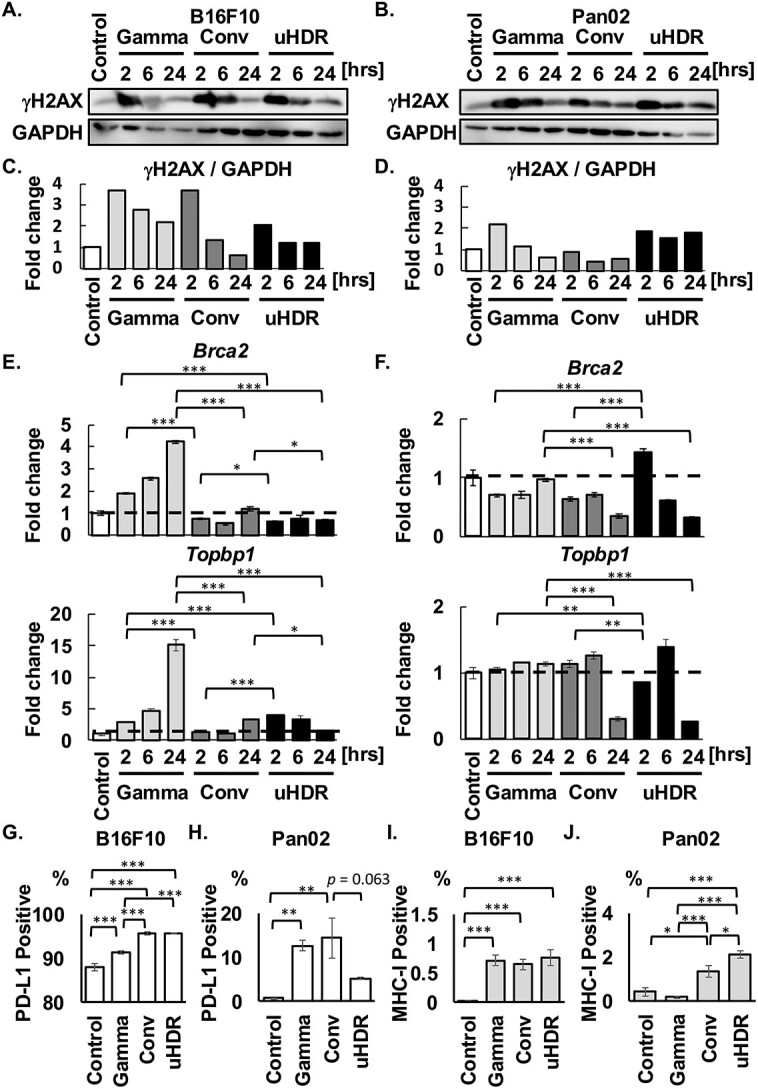
The impact of uHDR C-ion on DSB repair pathway. (A-D) γH2AX and GAPDH were shown, and GAPDH is the same loading control in Fig. 1. The ratios between γH2AX and GAPDH were shown. (E, F) Fold expression changes of *Brca2* and *Topbp1* were normalized with the control group in both cells. The dashed lines indicated in the bar graphs mean the value of 1, the same as the control. Data are indicated mean ± S.E. (G-J) The population of PD-L1-positive and MHC-I-positive cells were shown. Data are indicated mean ± S.E. ^*^P < 0.05, ^**^P < 0.01, ^***^P < 0.0001.

To investigate the DNA double-strand break (DSB) repair genes as another cellular response after γ-ray and C-ion, γH2AX was evaluated by western blotting. Figure 2A–D showed that both γ-ray and C-ion regardless of dose rate produced the DSB at the early time point (within 2 hrs) then they were reduced time-dependent. Interestingly, its expression after uHDR C-ion irradiation looks still high at 24 hrs after irradiation in both cells.

These results suggested that DSB repair progression after uHDR C-ion may differ from that after γ-ray. Therefore, the expressions of DSB repair-related genes (*Brca2* and *Topbp1*) were investigated by RT-PCR. Figure 2E showed that the *Brca2* expression after γ-ray was increased in a post-irradiation time-dependent manner. However, its expressions after C-ion irradiation were lower than those after γ-ray irradiation in B16F10. Consistent with *Brca2* expression, the *Topbp1* expression also increased in a time-dependent manner. The result showed that DSB after γ-ray irradiation was repaired in a time-dependent manner, consistent with the upregulation of the DSB repair-related gene; *Brca2* and *Topbp1* [19]. On the other hand, in Pan02, the changes in *Brca2* and *Topbp1* gene expression after γ-ray irradiation were not observed compared with Control (Fig. 2F). Although the genes were not dramatically changed in the expressions in Pan02, *Brca2* was increased to 1.4-fold expression only under the uHDR condition at 2 hrs post-irradiation, then may lead to an increase in *Topbp1* gene expression to 1.4-fold change at 6 hrs post-irradiation (Fig. 2F). Notably, a decrease in these gene expressions 24 hrs-post C-ion irradiation was observed.

A recent study showed that PD-L1 expression was upregulated in Brca2-deficient cells after irradiation [20], therefore, we evaluated PD-L1 expression (Fig. 2G and H). Although PD-L1 expression on B16F10 cells was more than 85% at the unirradiated state, it was increased after irradiation, especially C-ion enhanced its expression regardless of dose rate (Fig. 2G). While PD-L1 on Pan02 cells after γ-ray and Conv C-ion irradiation was increased with significance, interestingly there was a trend to suppress PD-L1 expression by uHDR (*P* = 0.063 vs. Conv) (Fig. 2H).

Several studies regarding uHDR with photon, electron, and C-ion irradiation showed FLASH effect on murine muscle [21], lung [22], brains [23, 24], and pigs’ skin [25]. Some mechanisms have been postulated, including radical recombination [26–28], but the underlying mechanisms have been unrevealed. In the present study, we showed cell-dependent responses, including the change in the expressions of DSB repair-related genes and immunogenic protein.

In addition, MHC-I was measured by flow cytometry (Fig. 2I and J). A significant increase in the expression was observed after irradiation with γ-ray and C-ion regardless of dose rate in B16F10, however, only C-ion increased MHC-I expression in Pan02, moreover, uHDR C-ion induced significantly higher expression than Conv C-ion.

Taken together, the present study showed that uHDR C-ion irradiation potentially has greater impacts on ER and modulates the repair pathway, which would not influence cell survival (Fig. 1A and B), but this phenomenon was cell-dependent. Especially in Pan02, uHDR C-ion irradiation may yield a more immune-promoting environment, with low suppressive (PD-L1) and high immune-promoting proteins (CRT and MHC-I). The underlying mechanism making the difference between both cells remains unclear. To the best of our knowledge, this is the first study to show the immunogenic proteins’ expression after uHDR C-ion irradiation on murine cancer cells.
